# Reflection on leadership behavior: potentials and limits in the implementation of stress-preventive leadership of middle management in hospitals – a qualitative evaluation of a participatory developed intervention

**DOI:** 10.1186/s12995-021-00339-7

**Published:** 2021-11-29

**Authors:** Elena Tsarouha, Felicitas Stuber, Tanja Seifried-Dübon, Natalia Radionova, Susanne Schnalzer, Christoph Nikendei, Melanie Genrich, Britta Worringer, Maja Stiawa, Nadine Mulfinger, Harald Gündel, Florian Junne, Monika A. Rieger

**Affiliations:** 1 Institute of Occupational and Social Medicine and Health Services Research, Faculty of Medicine, University Hospital Tuebingen, Tuebingen, Germany; 2 Department of Psychosomatic Medicine and Psychotherapy, Medical University Hospital Tuebingen, Tuebingen, Germany; 3 Echt:Zeit Coaching, Esslingen, Germany; 4 Department for General Internal Medicine and Psychosomatics, University Hospital Heidelberg, Heidelberg, Germany; 5 Institute of Psychology, Work and Organizational Psychology, University of Duisburg-Essen, Essen, Germany; 6 Institute of Occupational, Social and Environmental Medicine, Centre for Health and Society, Faculty of Medicine, Heinrich-Heine-University of Düsseldorf, Düsseldorf, Germany; 7 Department of Psychiatry and Psychotherapy II, Ulm University, Guenzburg, Germany; 8 Clinic of Psychosomatic Medicine and Psychotherapy, University Hospital Ulm, Ulm, Germany; 9 Department of Psychosomatic Medicine and Psychotherapy, Otto von Guericke University Magdeburg, Magdeburg, Germany

**Keywords:** Mental health, Stress prevention, Stress-preventive leadership, Middle management, Intervention, Hospital, Qualitative research, Germany

## Abstract

**Background:**

Mental health and stress prevention aspects related to workplace in hospitals are gaining increasingly more attention in research. The workplace hospital is characterized by high work intensity, high emotional demands, and high levels of stress. These conditions can be a risk for the development of mental disorders. Leadership styles can hinder or foster work-related stress and influence the well-being of employees. Through leadership interventions, leaders may be encouraged to develop a stress-preventive leadership style that addresses both, the well-being of the leaders and of the subordinates. A comprehensive qualitative description of leaders’ experiences with interventions on the topic of stress-preventive leadership is yet missing in the literature. Therefore, we address leaders of middle management regarding the development of stress-preventive leadership styles through supporting interventions. The research questions are: How do leaders of middle management perceive their leadership role in terms of effectiveness in stress prevention? Which potentials and limits in the implementation of stress-preventive leadership are experienced?

**Methods:**

The study follows a qualitative research design and content analysis. We conducted individual interviews with leaders of middle management (*n* = 30) of a tertiary hospital in Germany for the participatory development of an intervention. This intervention, consisting of five consecutive modules, addressed leaders of middle management in all work areas within one hospital. After participation in the intervention, the leaders were asked to reflect on and evaluate the implementation of the contents learned within focus group discussions. Overall 10 focus group discussions with leaders (*n* = 60) were conducted.

**Results:**

The results demonstrate that leaders of middle management perceived potentials for a stress-preventive leadership style (e.g., reflection on leadership role and leadership behavior, awareness/mindfulness, and conveying appreciation). However, limits were also mentioned. These can be differentiated into self-referential, subordinate-related, and above all organizational barriers for the implementation of stress-preventive leadership.

**Conclusions:**

Some of the organizational barriers can be addressed by mid-level leadership interventions (e.g., lack of peer-exchange) or possibly by adapted leadership interventions for top management (e.g., lack of stress-preventive leadership styles in top level management). Other organizational limits are working conditions (e.g., staff shortage) that can only be influenced by health policy decisions.

**Supplementary Information:**

The online version contains supplementary material available at 10.1186/s12995-021-00339-7.

## Abstract (de)

**Hintergrund:**

Aspekte der psychischen Gesundheit und der Stressprävention am Arbeitsplatz Krankenhaus gewinnen in der Forschung zunehmend an Beachtung. Der Arbeitsplatz Krankenhaus zeichnet sich durch eine hohe Arbeitsintensität, eine hohe emotionale Belastung und ein hohes Stresslevel aus. Diese Bedingungen können ein Risiko für die Entwicklung von psychischen Beschwerden darstellen. Führungsstile können arbeitsbedingten Stress hemmen oder fördern und das Wohlbefinden von Angestellten beeinflussen. Durch Fortbildungsinterventionen können Führungskräfte ermutigt werden, einen stresspräventiven Führungsstil zu entwickeln, der sowohl das Wohlbefinden der Führungskräfte als auch das der Mitarbeitenden anspricht. Eine umfassende qualitative Beschreibung der Erfahrungen von Führungskräften mit Fortbildungsinterventionen zum Thema stresspräventive Führung fehlt bisher in der Literatur. Daher untersuchen wir Führungskräfte des mittleren Managements bei der Entwicklung eines stresspräventiven Führungsstils durch unterstützende Fortbildungsinterventionen. Die Forschungsfragen lauten: Wie nehmen Führungskräfte des mittleren Managements ihre Führungsrolle hinsichtlich der Einflussnahme auf Stressprävention wahr? Welche Potentiale und Grenzen werden bei der Umsetzung eines stress-präventiven Führungsstils erfahren?

**Methoden:**

Ein qualitatives Forschungsdesign mit einer inhaltsanalytischen Auswertung wurde umgesetzt. Es wurden Interviews mit Führungskräften der mittleren Führungsebene (*n* = 30) eines deutschen Klinikums für die partizipative Entwicklung einer Fortbildungsintervention durchgeführt. Die Fortbildungsintervention bestand aus fünf fortlaufenden Modulen und richtete sich an Führungskräfte der mittleren Führungsebene aus allen Arbeitsbereichen eines Krankenhauses. Nach der Teilnahme an der Fortbildungsintervention wurden die Führungskräfte gebeten, die Umsetzung der gelernten Inhalte zu reflektieren und zu evaluieren. Dies erfolgte im Rahmen von zehn Fokusgruppendiskussionen mit Führungskräften (*n* = 60).

**Ergebnisse:**

Führungskräfte der mittleren Führungsebene nahmen Potentiale für einen stress-präventiven Führungsstil wahr (z. B. Reflexion der Führungsrolle und des Führungsverhaltens, Vermittlung von Wertschätzung). Ebenso wurden Grenzen für die Umsetzung einer stress-präventiven Führung thematisiert, die sich gliedern lassen in selbstbezogene, mitarbeiterbezogene und vor allem auch organisationale Barrieren.

**Schlussfolgerungen:**

Manche der organisationalen Barrieren können in Fortbildungsinterventionen für die mittlere Führungsebene adressiert werden (z. B. fehlender Peer-Austausch) oder möglicherweise in angepassten Fortbildungsinterventionen für das Top-Management (z. B. fehlende stress-präventive Führungsstile auf höheren Führungsebenen). Andere organisationale Grenzen sind Arbeitsbedingungen (z. B. Personalschlüssel) die nur durch gesundheitspolitische Entscheidungen beeinflusst werden können.

## Background

Work in hospitals is characterized by high work intensity and high emotional demands [1]. Beside numerous other psychosocial factors, social relationships and leadership are particularly relevant for the work context at hospitals [2]. Experiencing support from their leaders and fair leadership styles can act as protective factors for the mental health of subordinates [3]. Negative leadership styles of superiors, on the other hand, are risk factors for work-related stress among subordinates [4]. The major role of leaders for implementing health prevention [5] and their influence on subordinates’ health have been highlighted in numerous publications [6, 7]. Leaders are exposed to high work-related demands and face contradictory requirements: on the one hand, economic goals have to be achieved for the good of the company; on the other hand, they are required to lead in a stress-preventive manner to reduce the work-related stress of subordinates, which also has an economic aspect, since healthy workers may be less absent due to sick leave and tend to remain longer with their employer [8].

Different leadership tasks can hinder each other, as leaders may perceive health risks of their subordinates, but may not react preventively to them in order to achieve given short-term company goals [9]. The implementation of preventive measures, such as the risk assessment of subordinates’ mental stress by leaders of middle management, depends on the prioritization of the issue at higher management level [10]. Top management priority setting helps leaders of middle management weigh up different and sometimes conflicting corporate goals [10]. In our article top management includes, e.g. division leaders, chief physicians as well as board leaders.

According to Franke et al. ([11], see also Elprana et al. [12]) stress-preventive and health-promoting leadership comprises four ways in which leaders can influence their subordinates’ health: own overstrain and perceived stress, leadership behavior, shaping of working conditions and role model function. Elprana et al. [12], for example, describe those ways as follows: First, the leaders’ own strain can influence their attention and may reduce the support they give to subordinates. Second, supporting leadership behavior and conveying appreciation can have a positive effect on the well-being of subordinates; destructive leadership behavior can have a correspondingly negative effect [12]. Third, the design of working conditions by leaders, such as scope of action and decision-making or clear prioritization, can have an indirect impact on the health of subordinates; and finally, leaders who behave health-consciously can act as role models and authentically share their knowledge [12].

In studies on health-oriented leadership, the mental health of leaders themselves often takes a back seat, leaving far-reaching gaps in research [7, 13]. Depending on their scope of action, leaders can shape working conditions and promote their own health and the health of their subordinates [9]. However, this also implies that leaders must be aware of their own self-care as well as of their care for subordinates. Leadership concepts that focus on health of the employee have already been described in the existing literature. Three of the well-studied models of health-promoting leadership are Transformational Leadership [14], Leader-Member Exchange (LMX; [15]) and Health oriented Leadership (HoL; [16, 17]). The HoL concept comprises two constructs, self-directed health-promoting leadership (i.e. SelfCare) and subordinates-directed health-promoting leadership (i.e. StaffCare; [17]). SelfCare is considered an internal resource that enables a person to promote or protect their own health. SelfCare of the leader is the basis for a health-promoting leadership style (StaffCare; [17]). In order to implement StaffCare, leaders need to understand their responsibility for their subordinates’ health and recognize that they have an influence on the working conditions that affect subordinates’ health [18].

Recent studies show that leaders in hospitals are aware of work-related psychosocial factors and the resulting burdens on their subordinates, and make efforts to reduce these burdens [19–21]. Through leadership interventions, leaders can be supported in reflecting on their leadership role, in training methods and techniques of health-promoting leadership, and in shaping working conditions for themselves and their subordinates [22, 23]. As a comprehensive and detailed qualitative description of leaders’ experiences with such intervention, and whether and how they implemented stress-preventive measures is still missing from the literature, it will be addressed in this study.

This study originates from one subproject (Förderkennzeichen: 01GL1752C) of the transdisciplinary research network SEEGEN funded by the German Federal Ministry of Education and Research [24, 25]. The aim of the collaborative project is to develop and evaluate a complex intervention for health promotion in the hospital setting. The project is divided into 8 work packages (WP). This paper is a result of the WP 1C, which developed and evaluated an intervention in a participatory way for leaders of the middle management from all professional groups working in hospitals (e.g., physicians, nursing staff, administrative staff, IT etc.). The aim was to improve stress-preventive leadership skills to reduce psychosocial health risks amongst employees (leaders and subordinates). In this paper we present results on mid-level leaders’ perception of their influence on work-related psychosocial factors at the workplace hospital. Against the background of one’s own sandwich position [26], the possibilities and limits of stress-preventive leadership are reflected upon. The research questions of the article are:

- How do leaders of middle management perceive their leadership role in terms of effectiveness in stress prevention?
- Which potentials and limits in the implementation of stress-preventive leadership are experienced by leaders of middle management?

## Methods

### Study design and participants

**Fig. 1:**
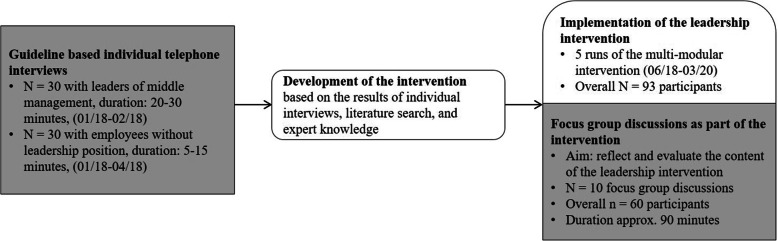
Study Design and Participants

The overall study of WP 1C was designed as a mixed-method approach with a comprehensive collection of qualitative data (see Fig. 1). However, this article focusses on the reflection of leaders of middle management in hospitals regarding their leadership role and their stress-preventive leadership behavior. Results of an additional quantitative approach will be published later (results of the additional standardized evaluation). For the participatory development of the intervention, leaders of the middle management (*n* = 30) of a tertiary hospital in Germany were interviewed. The interviews were analyzed content based. The aim was to identify needs in advance of the development of an intervention on stress-preventive leadership. Based on the results of these interviews, on results of additional interviews with employees without leadership responsibilities (*n* = 30) and further consideration of relevant literature, an intervention was developed. The intervention was conducted for leaders of middle management of different professional groups, e.g. physicians, nursing staff, therapeutic professionals, administration staff, IT staff, clinical services, office assistants, scientists, and other professions. The intervention began in June 2018 in the same hospital where the interviews and the standardized survey [27] were conducted, and was divided into five modules with content on SelfCare, recognizing stressors, leadership style, communication and team processes [28]. Each module was carried out with a temporal distance of two weeks to each other; between the fourth and fifth module, there was a larger break of three months in order to apply the contents learned in everyday work. There have been five runs of the intervention until March 2020. Focus group discussions were embedded in these runs. Ten semi-structured focus group discussions were conducted during the last (fifth) workshop module of the developed intervention and participating leaders were asked to reflect and evaluate the implementation of the learned contents. The data were analyzed by content analysis. In this article, only results from interviews with leaders during participatory development of the intervention (*n* = 30) and from the focus group discussions (*n* = 10) are presented.

### Ethical approval and consent to participate

Participation in the single interviews and focus group discussions was voluntary and consent was given by all participants. Participation in the single interviews could be revoked at any time. During the focus group discussions, the discussion participants were able to mark “off records” comments that would not be transcribed. Ethical approval for this study was obtained from the Ethics Committee of the Medical Faculty, University Hospital of Tuebingen (reference numbers: 622/2017BO2 and 208/2018BO1).

### Data collection

The semi-structured interviews with leaders were conducted with senior physicians (*n* = 15) and senior nurses (*n* = 15). The participants were recruited from a pool of leaders of middle management that had participated in other leadership interventions in the past, offered by the hospital’s Academy for Education and Personnel Development. They were contacted via e-mail by staff members of the Academy for Education and Personnel Development. Afterwards, a follow-up telephone call was made by FS. The interviewees were informed about the aim and content of the study. In order to participate, it was required to hold a leadership position at middle management. In agreement with the Employees’ Council, no other characteristics of the respondents were collected for data protection reasons. FS conducted the interviews via telephone. The participants were interviewed at their workplace. An interview guide was used for the interviews. This interview guide was developed by an inter-professional team of physicians and psychologists and focused on topics such as subjective perception of relevant aspects of stress-preventive leadership, required contents of a stress-preventive leadership intervention, and preferred intervention format. The questions of the interview guide are listed in Additional file 1. The interviews were recorded audibly, transcribed verbatim [29], and anonymized.

In addition to the interviews, focus group discussions were realized in the further course of the project; these focus group discussions were embedded in the actual implementation of the developed intervention. The participants of the intervention were informed that the intervention was part of WP 1C of the SEEGEN collaborative project and that the developed intervention, in which they were taking part, would be embedded in a complex intervention in the future. Furthermore, the leaders were informed about the further processing of the intervention in the overall project. The intervention was embedded in the official course offering of the Academy for Education and Personnel Development and took place during working hours of the participants. The focus group discussions were conducted by the two trainers, a psychologist and an educator (TS, SuS), who also, together with FS (psychologist), conducted the intervention. TS and SuS were trained beforehand in leading focus group discussions via a train-the-trainer session. All *n* = 60 leaders who took part in the last module (fifth module) of the intervention series in the facilities of the Academy for Education and Personnel Development also took part in one of the focus group discussions. To achieve an appropriate group size [30], the participants of each final intervention module were randomly divided into two roughly equal sized groups. A total of 10 focus group discussions with *n* = 5 up to *n* = 7 participants took place. To conduct the focus groups, a semi-structured interview guide was developed by FJ, FS and TS. The interview guide is available as Additional file 2. It contained questions on behavioral changes after participation in the intervention and reflections on the contents of the workshops. The discussions were conducted in two parts, with a 10-min break after about 45 min, followed by another exchange for about 45 min. Each focus group began with a 10-min self-reflection during which participants received all interview questions and were able to think about them and make notes. In the following 35 min the actual discussion took place which was recorded audibly, transcribed verbatim [29], and anonymized.

### Data analysis

The MAXQDA 2018 software was used to organize the data of the interviews and the focus group discussions during the analysis [31]. The data was analyzed using qualitative content analysis according to Mayring [32]. The analysis steps included the coding of the material applying a coding guideline, paraphrasing of the coded contents, and abstraction of the paraphrases using generalizations with the aim of reducing and structuring the data material. The coding guideline for the interviews as well the coding guideline for the focus group discussions were developed by FS and ET with the support of student assistants. The coding guidelines included category definitions, anchor examples and coding rules [33].

For the analysis of the interviews, a mainly deductive category system was used, which was further inductively differentiated or supplemented based on the data material. The deductively applied categories were based on the objectives of the interviews, such as the views, experiences and proposed solutions to concrete determinants of mental stress of health care workers in everyday life as well as the middle management leaders’ influence on it. Overall, there were 13 main categories (e.g., *stressors of leaders, stressors of subordinates, useful or needed content for the intervention, intervention conditions*), one of them further differentiated into subcategories. The main category *intervention conditions* was further differentiated into subcategories such as: *intervention format, didactic methodology, time scope* etc.

The first four transcripts of the focus group discussions were coded completely. The remaining transcripts of the discussions were reviewed completely but only new content was coded. Since it was not necessary to count the frequency of certain statements in order to answer the research questions, our approach does not lead to a significant loss of information. The aim of the analysis was to record the variety of relevant aspects for stress-preventive leadership by the participants.

In the analysis of the focus group discussions we applied a deductive category application. The categories were based on the theoretical background of the content of the intervention and the interview guide. The questions of the interview guide were formulated narratively. Since participants were encouraged to discuss their experience in a complex way with interwoven narratives, the categories were defined more broadly, and larger sense units were used as coding units. Overall, there were 6 categories. The categories were named *changes by and for the leader, changes on the side of the subordinates, difficulties in implementation, influence and effectiveness of the leader, relationship-oriented leadership style and implemented measures*. The contents of the categories were further differentiated and structured while paraphrasing and generalization. For this article, particular attention was paid to the contents of the categories *difficulties in implementation, influence and effectiveness of the leader* as well as *changes by and for the leader.*

The analysis steps (coding, paraphrasing, abstracting by generalizations) were carried out by at least two persons in order to achieve a high quality of analysis, e.g. through intersubjectivity [34]. Three researchers from the disciplines of sociology (ET, NR) and psychology (FS) and four student assistants were involved in the analysis of the described data. The reflexivity [35] was increased by additional data sessions with MR (occupational medicine), FJ (psychosomatic medicine) and TS (psychology). Thus, the analysis was enriched by critical feedback on occupational health and professional aspects. All quotations presented were translated from German into English (ET, TS) during the preparation of this article to provide readers with access to the original data. Taking into account the fact that concepts or words have different meanings in different languages [36], we focus on the overall content and meaning of the collected data in accordance with the applied analysis method [37].

The report of methods and results of this study follows the COREQ checklist [38].

## Results

The results are derived from the data of the individual interviews before and focus group discussions with leaders of middle management of a hospital in Germany after the intervention and are presented together. We focus on the results of the focus group discussions. These are enriched by selected results of the individual interviews for a deeper understanding of potentials and limits in the implementation of stress-preventive leadership. The quotes are given with the respective source attributions to the individual interviews or to the focus group discussions. Within single quotes there may be several aspects addressed, so that the quotes may be used also elsewhere within the results section for illustration. This illustrates the complexity and the interaction of various work-related factors in the hospital setting.

The participants described various barriers in the practical implementation of stress-preventive leadership and supportive measures within their scope of action. Barriers to the implementation of stress-preventive leadership in general, but also regarding specific measures from the intervention were reported. In connection with the barriers mentioned, some requirements for the implementation of a stress-preventive leadership style were explicitly mentioned, others can be derived. The barriers mentioned can be divided into the three areas leader-related barriers, subordinate-related barriers, and organizational barriers. Examples for these three areas are presented first. Afterwards, selected examples illustrate further specific barriers in the sandwich position, perceived scope of action regarding supportive measures as well as requirements for a stress-preventive leadership style on middle management.

### Leader-related barriers, subordinate-related barriers and organizational barriers

The barriers related to leaders include available resources (e.g., exhaustion of the leader), personal skills (e.g., recall of learned contents, discipline) and work practices (e.g., insufficient breaks). Leaders expressed that the implementation of stress-preventive measures in day-to-day work would become difficult over time. Although the positive effects of breaks and finishing work on time are known, they were hindered, e.g., by the discipline of the individual and by a lack of awareness (Quote 1).

***Quote 1 – Focus group discussion:*** *“Unfortunately, taking breaks doesn’t always work. Punctual closing time does not work at all right now [due to a] wave of sickness. But in between it worked quite well. I realized I need to work on myself a little bit better. It is dangerous that this is quickly lost in everyday life if you do not make yourself aware of it.”*

Stress-preventive measures for leaders in sense of SelfCare, which were part of the intervention, could be forgotten in moments where the suffering was not so great (Quote 2).

***Quote 2 – Focus group discussion:*** *“[Measures] that I had to implement on my own, [such as the One-Moment-Meditation], it was rather the problem that whenever I didn’t suffer [ …]*, *I quickly forgot [to apply them].”*

The implementation of stress-preventive leadership measures could also become more difficult due to subordinate-related aspects e.g., insufficient team orientation of subordinates and their willingness to be integrated into the team (Quote 3).

***Quote 3 – Focus group discussion:*** *“Nevertheless, if the team is big enough, you always have someone with you who pulls out, and that’s exactly the point where it’s very, very difficult to remain team-oriented and somehow do the right thing, the best thing for the whole team. If you have some who don’t really want to actively integrate themselves into the team. [ …] And getting someone like that into the team is often difficult.”*

Organizational barriers result from the given working conditions of clinical care and the organizational design of the workplace hospital. They include aspects such as staff shortages, unscheduled staff absences, large teams, or work intensity. These aspects were addressed as secondary themes in some of the selected quotations (e.g. Quotes 1, 3, 10, 11, 14, 16) and can therefore be taken as examples of organizational barriers. Furthermore, various leadership hierarchies are established in the organizational structure of hospitals, so that the scope of action for leaders of middle management is perceived as limited, e.g. with regard to team-oriented leadership (Quote 4).

***Quote 4 – Focus group discussion:*** *“We want team-oriented leadership. For team-oriented leadership we need a team structure and not a hierarchy. At the moment when there is a top-down hierarchy and when this is lived every day in the executive in such a way that one individual has the absolute ultimate decision-making authority, I don’t even need to start with team structure, at least with regard to certain processes that need to be changed.”*

### Specific barriers in the sandwich position

Some challenges for leaders of middle management were mentioned in the interviews with senior physicians and senior nurses and during the focus group discussions. For example, leaders of middle management emphasized a lack of exchange with other leaders (Quote 5).

***Quote 5 – Interview:*** *“As a leader you are often alone. Even though I talk things over with my senior nurse or with another leader, which I already do because I know two leaders in my group of acquaintances or friends - thank God. But I realize I really miss that as support.”*

Additionally, an increased work intensity due to the own sandwich position was mentioned. Demands and work-related pressure would be addressed to leader of middle management by top management and by subordinates. Top management would demand the implementation of structural requirements, while subordinates would become dissatisfied if difficulties arose during implementation (Quote 6).

***Quote 6 – Focus group discussion:*** *“I’m in a sandwich position, which means I have my supervisor on top of me and the team below me. I get pressure from above to implement a clear structural specification and pressure from below, by the team: No, you can’t do it that way. Or I experience dissatisfaction [in the team] directly, as I also work at the base on some days.”*

Participants also stated that work-related pressure and stress would be passed through several hierarchical levels to the lower levels of management (Quote 7).

***Quote 7 – Interview:*** *“I realize that my supervisor is under a lot of pressure from her leader and that this pressure causes her stress. She then passes on this stress.”*

### Scope of action regarding supportive measures

The leaders interviewed perceived limits in terms of organizational structure and saw potentials to influence given psychosocial demands through appropriate leadership behavior. In terms of perceived influence, participants distinguished between situational measures and future-oriented possibilities of regulation. Situationally, leaders of middle management could support their subordinates in patient care, whereby they could cause additional stress for themselves due to the associated extra work. According to the interview partners, work-related stress for subordinates could be avoided or reduced with a sustainable planning of the duty roster (Quote 8).

***Quote 8 – Focus group discussion****: “I have experienced the point as at least ambiguous in day-to-day [work]. There are immediate methods how I can help to minimize stress and those that are more future-oriented and will pay off in the medium term. For example, when I say OK, I’m going to work with you and take the next patient [ …]*. *This means, however, that I reduce stress on [the side of the subordinates] by creating additional one for myself, because this keeps me away from my other tasks and my to-do list. But then there are also control options. We receive the duty roster for review before it is released to see if we still have ideas, so I can have some influence on it.”*

The participants reflected the intervention contents also concerning the leadership behavior of the top management and noticed divergences. During the discussions, it was stated that the top management would not exercise a stress-preventive leadership. This aspect is described in more detail in the section “Requirements across hierarchical management levels”. Furthermore, it was explicitly stated that a situation-related stress-avoiding behavior of leaders of middle management towards subordinates could lead to an additional burden on leaders. Participants recognized that this additional burden must have limits and feedback of the burden must be communicated to the top management (Quote 9).

***Quote 9 – Focus group discussion:*** *“So many times I asked myself: what are our leaders doing? They really don’t do much of what we have been taught here. And you try to optimize the situation for your subordinates. It is always at my expense, without exception. We were understaffed at one point and as a senior physician you support the routine work and do patient care. [Afterwards] you do your main work in the evenings until midnight [ …] You have to seek the dialogue with your leader because you can’t work in the red zone all the time. [ …] I realized that very clearly through the intervention.”*

The participants emphasized several times, both in the individual interviews and in the focus group discussions, that stress-preventive leadership was also dependent on staffing ratio. Inadequate staffing could mean, for example, that in the event of absences due to illness, substitution by other employees was necessary, or understaffed shifts result. At the same time, the leaders of middle management perceived no opportunity to influence the staffing ratio. In the case of increased work intensity due to an insufficient number of staff, one possibility for leaders to prevent stress could be a conscious relationship-oriented leadership style, in which subordinates are shown appreciation for their willingness to take on stand-in duties (Quote 10).

***Quote 10 – Focus group discussion****: “What runs counter to the team concept: We have a tight staffing and as soon as one drops out, it’s patchwork and then you try to make it work somehow. The same people always step in and don’t refuse when they are asked. I can only partially change that, it’s not in my power. I am not responsible for the sickness absence and I am also not responsible for the staffing ratio and I cannot change anything about it. But I can notice the persons who always step in and who always say ‘yes’ and I can also mirror that, and that helps.”*

In addition, a clear prioritization of tasks with subordinates and the communication of this prioritization to members of other sections or to the top management were mentioned. The responsibility for setting priorities towards third parties was assumed by the participants in their role as leaders, e.g. if tasks were not carried out (Quote 11).

***Quote 11 – Focus group discussion:*** *“Over the summer, I had one subordinate less. [ …] We all sat down together, and I, as team leader, said that certain tasks would simply remain undone, and that’s it. [ …] When someone else came and complained about it to my team, I stood up and said: you can take over these tasks yourself, you don’t need us to do that and we are currently understaffed.”*

The prioritization of tasks by the leaders of middle management and the skipping of certain tasks seemed to be easier if they were supported by the top management. In contrast, leadership hierarchies could limit the setting of priorities by the leaders of middle management and hinder stress-preventive leadership if suddenly different priorities were set (Quote 12).

***Quote 12 – Focus group discussion:*** *“I had made a note of the fact that, at short notice and from the outside, i.e. the head of department or others, laws or changed priorities make the whole thing incredibly difficult (agreement). You have made a plan, you have a weekly plan, you only have a daily plan or a morning plan and a call comes in: our priorities are from now on and for the next two weeks … boom.”*

After participating in the intervention, leaders reflect on the compatibility of demands of the top management and a stress-preventive leadership style. Participants indicated that they would like to give a higher priority to the concerns of their subordinates (Quote 13).

***Quote 13 – Focus group discussion:*** “*If the head secretary’s office calls with a matter [from time to time], then I just reflect on [the prioritization] even more and think to myself, why is this now the first priority and the people I’m with every day, why do I put [their matters] on hold. Now I think: o.k. even when those from the head secretariat call a third time because the matter has not yet been settled, then I do not care. The matters on ward are [considered] first.”*

### Requirements across hierarchical management levels

A special characteristic of middle management became apparent in the role model function of the top management and the dependence on top management and decision-makers (Quotes 4, 12). In the focus group discussions, participants expressed a discrepancy between the content learned about stress-preventive leadership style and the behavior of the top management (see also Quote 9). They saw no possibility of influencing the awareness and implementation of a stress-preventive leadership style of their division leader. However, the reflection and awareness of a stress-preventive leadership style of the divisional leader is seen as important in order to implement self-referential stress-preventive working practices (SelfCare) in the middle management and, e.g. carry out breaks (Quote 14).

***Quote 14 – Focus group discussion:*** *“An important issue for me was that I actually don’t have much scope of action [ …]. Our divisional leader makes all the mistakes that can be done in terms of stress. It starts with the duty roster [ …]. There are no conversations in a calm atmosphere. We have a high stress level during work, so breaks cannot be taken. [ …] I do it like you do now, when I run through the corridors, then I consciously make myself walk slowly and take a deep breath. And then I do one thing at a time. I have now become very aware of the fact that my divisional leader does not [ …] do the staff care that is actually necessary. And I cannot do anything about it as long as he [divisional leader] is not aware of it himself. I wish he would participate in this course.”*

Stress prevention would also require that agreements between hierarchical levels on work procedures are adhered to (Quote 15).

***Quote 15 – Focus group discussion:*** *“Interviewee (I): For me, stress prevention includes structure and maintaining agreements. So when the chief physician […] consults with his senior physicians that rounds are always at [the same time] and it will be decided who can be sent home the next day and the reports are ready then […] - and*

*I: Mostly it is not that way.*

*I: And then nobody does [what was agreed upon].”*

The example of time management illustrates that certain work practices occur across hierarchical levels and could cause stress. Leaders of top management would place orders without sufficient time budget to leaders of middle management. This insufficient time management would in turn be passed on to their subordinates. And consequently, it would be accepted that tasks would also be processed at weekends or after working hours (Quote 16).

***Quote 16 – Interview****: “We need to think about a general communication structure within the hospital. This is not well practiced from above. Tasks are set without sufficient time budget. And you tend to behave in the same way and to say: Here you have a subtask. And the other one says: when should I do it? And then you say: just like me on the weekend or in the evening. That is not a good answer.”*

Appropriate time management was perceived as a stress-preventing working practice and as a leadership task. Subordinates would like to have appropriate time management at work (Quote 17).

***Quote 17 – Interview:*** *„I think subordinates would like the leader to be able to estimate realistically how much time a particular task requires. I think this is regularly misestimated.”*

Repeatedly, it became clear in the conversations that the topic of stress-preventive leadership style should also be addressed at higher levels of management. This would be decisive for the implementation of stress-preventive leadership at subordinate management levels. The need was formulated that contents from the discussions of the intervention should be passed on to the top management so that they could become aware of the requirements of a stress-preventive leadership culture and react accordingly (Quote 18).

***Quote 18 – Interview:*** *“It should not be just an intervention for leaders [of middle management]. Each participating group should work out a few things that are communicated to higher management. That it is not only something for us, but that they also receive feedback on a few key points that occur frequently in the courses. So they may be able to react and benefit from it.”*

## Discussion

The aim of the study was to describe potentials and limits in the implementation of stress-preventive leadership of middle management in hospitals. Leaders of middle management perceived their leadership role in terms of effectiveness as limited. The results demonstrate that our participants perceived not only self-referential and subordinate-related barriers, but, above all, organizational limits in the implementation of stress-preventive leadership. Leaders of middle management missed the support of an exchange with colleagues at their management level within the hospital. Due to the sandwich position, work-related pressure was felt, not only from the top management but also from the subordinates. Pressure would also be transmitted through the various hierarchical levels of management to the subordinates. After the intervention, participants mentioned opportunities to shape the working conditions of their subordinates. Leaders expressed that they could provide situational relief for their subordinates, but also take plannable preventive measures. These opportunities could have implications in terms of reducing perceived mental stress of leaders of middle management. Short-term support in patient care was described as an additional task and additional burden for leaders. In addition, participants reflected that their scope for shaping good working conditions for themselves (SelfCare) and for their subordinates [StaffCare; 17] depends on the leadership behavior and the sense of responsibility for a stress-preventive leadership style at top management level. Burdens for leaders of middle management should be communicated to the next higher management level, as should the requirements for creating good working conditions. Stress-preventive work practices (e.g., binding agreements or sufficient time management) should be addressed across hierarchical management levels.

**Fig. 2:**
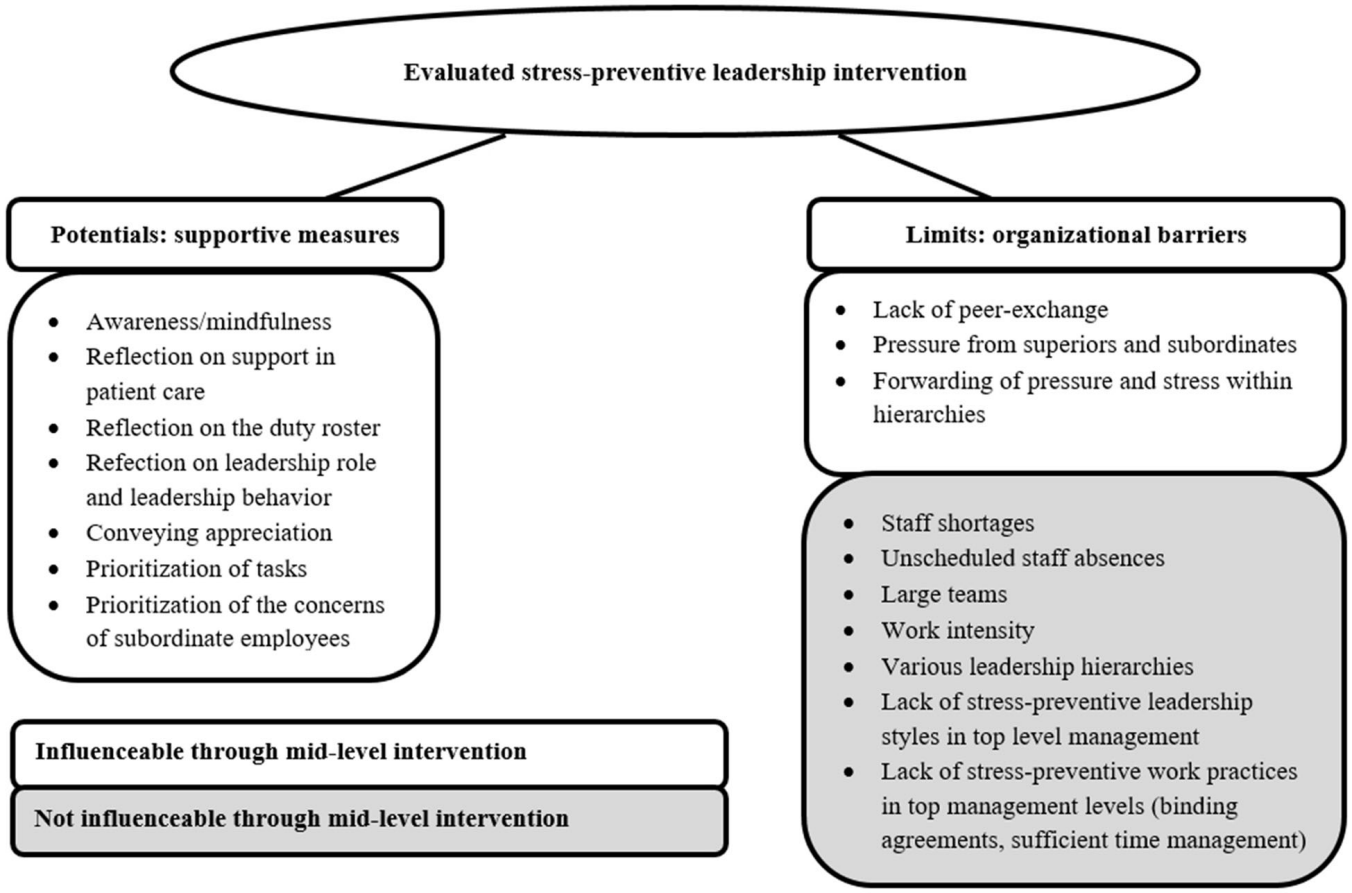
Perceived Potentials and Limits of the Intervention on Stress-Preventive Leadership

In the results section we described organizational barriers, supporting measures and perceived requirements for the implementation of a stress-preventive leadership style. From the perspective of participating leaders these aspects can be influenced differently in the context of a mid-level intervention such as the one this study is based on (see Fig. 2). All mentioned supportive measures can be reflected upon and corresponding techniques can be taught in interventions. In contrast, some organizational barriers may not be directly influenced by such an intervention. Since the stated requirements are relevant or have an impact across hierarchies, these cannot be sufficiently influenced in the context of an intervention that is directed exclusively at leaders of middle management. Regarding the second research question, leaders of middle management experienced potentials and limits in the implementation of stress-preventive leadership as described below.

### Potentials of mid-level interventions

Reflection on one’s own leadership role and leadership behavior, and the awareness of the leadership styles and leadership behavior of the top management are to be evaluated as a result of the intervention participation. Our participants reported that self-referential mindfulness may be neglected in the absence of distress. Mindfulness is understood as present-moment awareness with an observing, non-judging stance and leader mindfulness is significantly related to subordinate well-being [39]. In addition, an increased awareness of the leader may also lead to a stress-preventive leadership towards subordinates (StaffCare), e.g. by preventing stress from being passed on to other employees or that more attention is paid to the well-being of their subordinates [12]. By participating in the intervention, leaders recognized that they can influence the working conditions of their subordinates in a stress-preventing way by adopting a relationship-oriented leadership style [14, 15, see also relational coordination, Gittell et al. [40]. They did this, for example, by conveying appreciation to their subordinates. Appreciation as a cornerstone of Transformational Leadership can lead to higher job satisfaction among subordinates [41]. Transformational Leadership affects the perceived working conditions and leads to “employees experiencing their work as meaningful, having influence and being involved in their work” [42].

A given work intensity was also handled through prioritization of tasks and the prioritization of the concerns of subordinates, which could lead to stress prevention among subordinates. Several prioritization strategies [43, 44] could be part of an intervention. On the one hand, the support of the higher superior leader was beneficial for the prioritization of tasks for oneself and towards subordinates. On the other hand, prioritization was limited by the given top-down hierarchy and corresponding decision-making powers.

### Limits of mid-level interventions

Some organizational barriers such as, e.g. staffing ratio, unscheduled staff absence, large teams, work intensity and a given top-down decision-making process cannot be directly influenced by interventions (see colored barriers in Fig. 2). Factors, such as insufficient staffing ratio or absence of personnel due to illness favor overtime and the absence of breaks [20]. In recent studies leaders see a connection between sickness absence of their subordinates and the extreme work stress in hospitals [19], which is exacerbated by a given shortage of staff [20]. In the event of short-term schedule changes due to sickness-related absences, leaders must guarantee appropriate recovery phases. The distribution of free days within the shift system, are important for recovery and health [45]. Given insufficient staffing, a health-oriented duty roster with reliable working and private times and a balanced substitute planning may be hardly achievable.

Studies have shown that the role model function of leaders in the hospital setting includes respect, appreciation, openness, and empathy [36]. Some of the intervention participants in our study stated that their leaders did not exemplify adequate stress-preventative leadership styles and thus, for example, made SelfCare for leaders of middle management more difficult. This would result in overtime and a lack of breaks. Studies have shown an association between long working hours and an impairment of mental health, and that sufficient breaks are necessary for recovery and stress compensation and have a positive impact on mental health [37]. Additionally, the participants in our study reflected on stress-enhancing and stress-preventive work practices associated with hierarchical management levels such as inadequate time management or binding agreements. Inadequate time management is perceived as a barrier to a stress-preventive leadership style, both in terms of SelfCare and StaffCare. Further interventions across hierarchical levels could provide key skills and improve job satisfaction by using time management strategies to increase productivity and reduce stress [38].

A challenge for leaders of middle management may be that requests are made without knowledge of the time requirements that already exist and that different activities are given the same importance and value [34]. Studies suggest that a tendency towards a higher burden on lower and middle management levels could be explained by the responsibility for operational tasks and the sandwich position of leaders [8]. In addition, perceived constraints on workplace design decreases with increasing hierarchical management levels [6]. We conclude that leaders of middle management are exposed to high job demands and at the same time have limited decision latitude (control). According to the Job Demand-Control Model, this can lead to psychosocial work-related strain [39]. In line with other studies, a need for institutionalized communication structures across hierarchical levels was identified [16]. In addition, our study also identified a lack of communicative exchange between leaders at the same management level.

### Strengths and limitations

To the best of our knowledge, this is the first study conducted in the context of a participatory development of an intervention on the topic of stress-preventive leadership style and there are only a few studies with a qualitative evaluation of an intervention for leaders in hospitals [46–51]. As a limitation of the study it can be mentioned that due to the requirement for anonymization of the participants by the Employees’ Council, it was not possible to consider the needs, barriers and measures implemented, taking into account the relevance of certain aspects for different professional groups and areas of work. Leaders of different occupational groups in hospitals are perceived differently by their subordinates and work processes differ [52], which may result in different demands on the respective leader. In the participatory development of the intervention, however, preferred contents and needs from the perspective of nurses and assistant doctors (i.e. subordinates) were included.

## Conclusions

Within the framework of (mid-level) interventions, leadership skills can be promoted [53] with regard to a stress-preventive and health-oriented leadership style [48], e.g. reflection on leadership role and leadership behavior, conveying appreciation, prioritization of tasks. Other preconditions for good working environments cannot be eliminated within the framework of mid-level leadership intervention (e.g., stress-preventive leadership styles and stress-preventive work practices across hierarchical levels), but require a more comprehensive intervention that also addresses top management. The need for leadership skills and the specific job demands in dependence of various hierarchical levels in everyday hospital life have not yet been sufficiently researched [54]. However, recent studies have shown that interventions of leaders in the field of mental health have an impact on health-promoting leadership behavior [55] and in addition, supportive leadership behavior has an impact on subordinates’ health [56]. In contrast, some barriers and preconditions cannot be eliminated by interventions at all, e.g., staffing ratio or work intensity. These are structural effects of health policy decisions. The present results can serve as a basis for political discussions among decision-makers in the health sector. Without changes in the framework conditions for the workplace hospital, interventions cannot achieve substantial improvements in terms of stress-preventive leadership and related health prevention [48].

## Supplementary Information

**Additional file 1.** Telephone Interview Guide

**Additional file 2.** Interview Guide Focus Group Discussion

## Data Availability

The Chief Executive Board and the Employees’ Council of the tertiary hospital had to approve the implementation of this study. This approval required that raw data be made available only to direct project members.

## Abbreviations

LMX: Leader-Member Exchange
HoL: Health oriented Leadership
WP: work package(s)
