# Supplementary material for: Reflection on leadership behavior: potentials and limits in the implementation of stress-preventive leadership of middle management in hospitals – a qualitative evaluation of a participatory developed intervention
Source: J Occup Med Toxicol. 2021 Nov 29;16:51. doi: 10.1186/s12995-021-00339-7 (PMC8628435; doi:10.1186/s12995-021-00339-7)
Supplement: Supplementary file 1 — Additional file 1. Telephone Interview Guide [file 12995_2021_339_MOESM1_ESM.pdf]

Additional File 1: Telephone Interview Guide

Telephone interview guide on the perspectives and preferences of leaders for an advanced intervention on "stress-preventive leadership"

Introduction

About the project in general:

- The interview we are conducting today is part of a project funded by the Federal Ministry of Education and Research entitled "Mental health at the workplace hospital".
- The Department of Psychosomatic Medicine and Psychotherapy would like to develop an advanced intervention for leaders on the topic of stress-preventive leadership of employees as part of this project.

About the following interview:

- That's why you are being interviewed as an expert today.
- We would like to learn about your perspective and needs for a future intervention on "stress-preventive leadership".
- There are no right or wrong answers. We are interested in your personal assessments.

Procedure:

- The interview takes about 20 minutes.
- It consists of 9 main questions on which further questions can be asked as needed to learn more about your perspective.
- I will record the interview on tape for later analysis.
- Would you be okay with that?

Anonymity:

- Our conversation recorded on tape is then converted from an audio file to a text file. All references to your identity will be deleted.
- After that there are no notes left regarding: your name, place of employment, assigned employees or other information related to your identity.
- The analysis of the data and its publications are completely anonymuous. We strictly take care that no conclusions can be drawn about your person. There is no analysis at individual participant level.
- Your participation is voluntary and can be terminated at any time.

Obtain consent:

- Do you agree with that?
- Do you have further questions in this regard?
- If further questions arise during the conversation or in case you have not understood something, please do not hesitate to ask.

Interview guide:

**Key questions**	Specific requests	Sustaining questions
**1. What comes to your mind spontaneously on the subject of "stress-preventive leadership"?**	1 a) How would you define/describe stress-preventive leadership? What does it include, what constitutes stress-preventive	

	leadership?<br><br>1 b) Is it possible to learn stress-preventive leadership and if so, what can you learn?<br><br>1 c) How would you describe your influence on the stress load of your employees?	
**2. Do you consider further intervention on the subject of "stress-preventive leadership" to be useful in principle?**	2 a) If yes, why? If not, why not?<br><br>2 b) Would you be interested in participating in such an intervention?	
**3. What would you like to learn in an intervention on "stress-preventive leadership"?**	3 a) Which contents would be relevant for you in an intervention on "stress-preventive leadership"?<br><br>3 b) Which job-related contents would be relevant for you as a leader?<br><br>3 c) Which work style and personality related contents would be relevant for you as a leader?<br><br>3 d) Which communicative and interaction-related processes would be relevant for you and your work as a leader?	Could you be more specific?
**4. What framework conditions would you prefer for an intervention on "stress-preventive leadership"?**	4 a) Which condition would you prefer?<br>For example:<br>- Presence group seminars,<br>- Individual coaching,<br>- Online modules	
**5. Which didactic methods would you prefer for an intervention on "stress-preventive leadership"?**	5 a) How would you like to get the contents communicated didactically? (e.g. through presentations, workshops, discussions, webinars, online chat panel)<br><br>5 b) How practical are	

	online modules in addition to face-to-face seminars?<br><br>5 c) What do you expect from the intervention leader?	
**6. How much time would be practicable for you as a leader to participate in an intervention on "stress-preventive leadership"?**	6 a) What extent, what frequency, what period of time would be compatible with your everyday working life?	
**7. What could an intervention on "stress-preventive leadership" specifically change in your department?**	7 a) What are your concrete wishes and goals?<br><br>7 b) What benefits would you expect from an intervention on "stress-preventive leadership"?<br><br>7 c) What could an intervention on "stress-preventive leadership" change in your own leadership behavior?<br><br>7 d) How would you notice at the end of the intervention that your expectations have been fulfilled?<br><br>7 e) What should not happen during the intervention?	
**8. Which changes would your subordinate employees like to see through the strengthening of the stress-prevention competence of their leaders?**	8 a) What are their specific wishes and goals?<br><br>8 b) How would your employees notice that you have attended an intervention on "stress-preventive leadership"?	
**9. In your opinion, is there anything important that we have not yet discussed?**		
