# Supplementary material for: Reflection on leadership behavior: potentials and limits in the implementation of stress-preventive leadership of middle management in hospitals – a qualitative evaluation of a participatory developed intervention
Source: J Occup Med Toxicol. 2021 Nov 29;16:51. doi: 10.1186/s12995-021-00339-7 (PMC8628435; doi:10.1186/s12995-021-00339-7)
Supplement: Supplementary file 2 — Additional file 2. Interview Guide Focus Group Discussion [file 12995_2021_339_MOESM2_ESM.pdf]

Additional File 2: Interview Guide Focus Group Discussion

Focus groups to reflect the intervention “stress-preventive leadership in hospital”

Questions focus groups

Part one:

What has changed **for yourself** as a result of the intervention “stress-preventive leadership in the hospital“? How has it changed? Are there **example situations**?

What has changed for **your subordinates** through your intervention participation? How has it changed? Are there **example situations**?

How effective are you in **your role** as a leader in terms of subordinates’ stress prevention? And why? And why not? (**example situations**) Has something changed through your intervention attendance?

Part two:

What is **the most important thing** for you about relationship-oriented leadership? (**example situations**)

What was the most difficult thing in **implementing** relationship-oriented leadership? (**example situations**)

How could the **contents** of the single modules of the intervention be implemented in everyday life? What experiences have you had with it (pro and contra)?

Which **obstacles**, **barriers** (e.g. own, system, subordinates) have made the implementation of content aspects difficult? (**example situations**)
